# Genome of extreme halophyte *Puccinellia tenuiflora*

**DOI:** 10.1186/s12864-020-6727-5

**Published:** 2020-04-19

**Authors:** Rui Guo, Long Zhao, Kaijian Zhang, Dan Gao, Chunwu Yang

**Affiliations:** 1 Key Laboratory of Dryland Agriculture, Institute of Environment and Sustainable Development in Agriculture, Chinese Academy of Agricultural Sciences, Beijing, 100081 China; 2 Key laboratory of Molecular Epigenetics of Ministry of Education (MOE), Northeast Normal University, Changchun, 130024 China; 3 Institute of Genetics and Developmental Biology, Chinese Academy of Sciences, Beijing, 100101 China; 4 Beijing Novogene Bioinformatics Technology Ltd, Beijing, 100083 China

**Keywords:** Genome, Halophyte, Salinity, *Puccinellia tenuiflora*

## Abstract

**Background:**

*Puccinellia tenuiflora*, a forage grass, is considered a model halophyte given its strong tolerance for multiple stress conditions and its close genetic relationship with cereals. This halophyte has enormous values for improving our understanding of salinity tolerance mechanisms. The genetic information of *P. tenuiflora* also is a potential resource that can be used for improving the salinity tolerance of cereals.

**Results:**

Here, we sequenced and assembled the *P. tenuiflora* genome (2n = 14) through the combined strategy of Illumina, PacBio, and 10× genomic technique. We generated 43.2× PacBio long reads, 123.87× 10× genomic reads, and 312.6× Illumina reads. Finally, we assembled 2638 scaffolds with a total size of 1.107 Gb, contig N50 of 117 kb, and scaffold N50 of 950 kb. We predicted 39,725 protein-coding genes, and identified 692 tRNAs, 68 rRNAs, 702 snRNAs, 1376 microRNAs, and 691 Mb transposable elements.

**Conclusions:**

We deposited the genome sequence in NCBI and the Genome Warehouse in National Genomics Data Center. Our work may improve current understanding of plant salinity tolerance, and provides extensive genetic resources necessary for improving the salinity and drought tolerance of cereals.

## Background

Salinity stress affects over 6% of the global land area and is a severe problem that limits agriculture [1, 2]. Halophytes are remarkable plants that tolerate high salinity that would kill 99% of other plant species (glycophyte), and are applied to improve saline soil [3, 4]. Some extreme halophytes can survive salinity levels > 1000 mM NaCl, whereas glycophytes, such as rice and *Arabidopsis*, can only survive 50–100 mM NaCl [4, 5]. Most botanists believe that these salt-sensitive glycophytes may provide limited insights into mechanisms of salinity tolerance, and that extreme halophytes may have enormous values for improving our understanding of salinity tolerance mechanisms [4–6]. Given that many important crops are gramineous, understanding the salinity tolerance mechanisms of gramineous halophytes will be helpful in improving the salinity or drought tolerance of cereal crops. Although the genomes of several salinity-tolerant plant species have been reported [7–10], the genome of an extreme Gramineae halophyte is unavailable. *Puccinellia tenuiflora* (2*n* = 14) is a perennial halophyte of the Gramineae and is distributed in Asian and European grasslands [3, 11, 12]. It is a forage grass with high nutritional value and strong tolerance for multiple stress conditions, such as drought, disease, and chilling [3, 11, 12]. *P. tenuiflora* can survive at pH 10 and 900 mM NaCl [3, 11–14] and can grow normally and produce seeds under some extreme soil conditions (2–3% salt content and pH > 10) [14, 15]. Given these qualities, *P. tenuiflora* has been used to recover and exploit saline grasslands or croplands in northern China [14, 15]. A growing number of molecular studies have focused on *P. tenuiflora* [12, 16–28]. Currently, *P. tenuiflora* is recognized as a model halophyte [3, 12]. Unfortunately, the genomic sequence of *P. tenuiflora* is unavailable. Here, we provide first report on the *P. tenuiflora* genome. Our work may provide extensive genetic resources for improving the salinity or drought tolerance of cereals.

### Construction and content

#### Evaluation of genome size

**Fig. 1:**
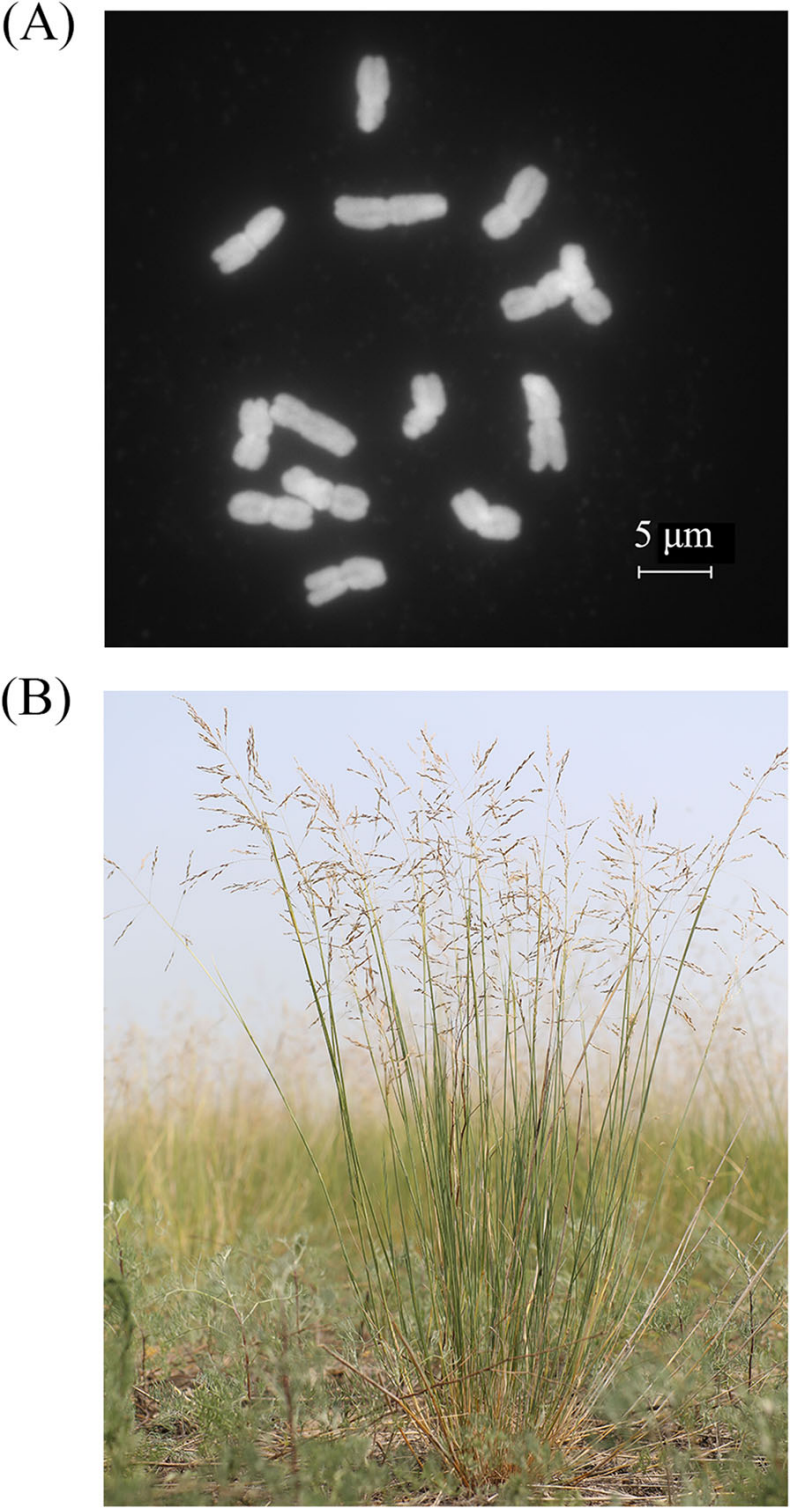
Chromosome number (**a**) and habitat (**b**) of *P. tenuiflora*

Taxonomy characteristics of *Puccinellia tenuiflora* are available at Flora of China (http://www.efloras.org/florataxon.aspx?flora_id=2&taxon_id=200026128). We surveyed the chromosome number of *P. tenuiflora* according to Kato et al. [29]. Total genomic DNA was extracted from fresh leaves. We used the conventional method to estimate the *P. tenuiflora* genome size. Briefly, we generated 49 Gb of high-quality short-insert Illumina reads to analyze the *K*-mer frequency of distribution [30]. Genome size was calculated using the following formula: Genome size = total *K*-mer number /*K*-mer depth [30, 31], in which *K*-mer depth is the peak value of *K*-mer distribution. The chromosome number of *P. tenuiflora* is 14 (Fig. 1). Our *K*-mer analysis showed that the genome size of extreme halophyte *P. tenuiflora* was 1.303 Gb (2n = 14) and the genome was complex, with 1.56% heterozygosity and 65.5% repeat content (Table 1).

**Table 1:** Results of *K-mer* analysis. The *K-mer* was defined as 17 bp to assess *P. tenuiflora* genome size by the following formula: total *K-mer* number/*K-mer* depth. The heterozygous ratio was determined by the number of heterozygous *K-mer/*total *K-mer* number

*K-mer*	Depth	n_*kmer*	Genome_size (Mb)	^a^Revised genome_size (Mb)	Heterozygous_rate (%)
17	31	41,192,925,796	1328.80	1303.06	1.56

^a^Excluded effects of uncorrected *K-mer*

### Genome sequencing

Illumina paired-end (PE) libraries were constructed with short insert sizes of 250 and 450 bp. Illumina mate-pair (MP) libraries were constructed with insert sizes of 2, 5, and 10 k bp (Table 2). We generated 209.13 Gb of raw data by the PE libraries, and 197.38 Gb of raw data by the MP libraries. The Illumina libraries were sequenced on Illumina HiSeq XTen platform. We also sequenced 56.12 Gb of PacBio long reads and 161.03 Gb of 10× genomics barcoded reads (Table 2).

**Table 2:** Raw data of *P. tenuiflora* sequencing

Libraries	Insert size	Total data (Gb)	Sequence coverage (X)
Illumina reads	250 bp	122.03	93.87
	450 bp	87.1	67
	2 kb	70.29	54.07
	5 kb	51.3	39.46
	10 kb	75.79	58.3
PacBio reads	20 kb	56.12	43.17
10× Genomics		161.03	123.87
Total	–	623.66	479.74

### Genome assembly

Because the *P. tenuiflora* genome is highly complex and repeated, its genome was assembled by a combined strategy of PacBio (third-generation), 10× genomic technique, and Illumina Hiseq (second-generation). We generated 312.6× reads of Illumina, 43.2× read of PacBio and 123.87× reads of 10× genomic. First the PacBio sequences were corrected for errors. The accurate sequences of PacBio were assembled into primary contigs based on FALCON (Branch 3.1) [32] and FALCON-Unzip software (https://github.com/PacificBiosciences/FALCON_unzip). After treatment with FALCON-Unzip software, we corrected errors of these contigs using PacBio sequences based on quiver software [33] and using Illumina data based on pilon software [34], and finally obtaining consensus sequences of high quality. Next, we used Illumina long reads of 2, 5, and 10 kb to elongate and combine the preassembled contigs into scaffolds based on SSPACE software [35], and then used 10× genomics linked-reads to further elongate and combine the scaffolds based on 10× FragScaff software. Lastly, we used Purge Haplotigs software (https://bitbucket.org/mroachawri/purge_haplotigs/overview) to filter the redundant sequences caused by high heterozygosity. Finally, we assembled 2638 scaffolds with a total size of 1.107 Gb, contig N50 of 117 kb, and scaffold N50 of 950 kb (Table 3).

**Table 3:** Assembly results of *P. tenuiflora* genome

Sample ID	Length		Number	
	Contig (bp)	Scaffold (bp)	Contig	Scaffold
Total	1,095,388,111	1,107,157,923	14,036	2638
Max	803,180	7,202,224	–	–
Number > =2000	–	–	13,349	2183
N50	117,188	949,910	2936	338
N60	97,500	788,398	3958	465
N70	80,583	601,430	5194	625
N80	64,330	447,145	6714	839
N90	45,138	278,370	8711	1152

### Genome annotation

### Annotation of replicate sequences

Transposable elements (TEs) of the *P. tenuiflora* genome were annotated. We used two methods to find the TEs. The first method was RepeatMasker (version 3.3.0) to discover TEs in an integrated known replicate sequence library (Repbase 15.02) and the de novo replicate sequence library constructed by RepeatModeler (Version 1.0.5) [36, 37], RepeatScout [38], and LTR_FINDER [39]. The second method detected TEs in the *P. tenuiflora* genome using RepeatProteinMask by searching against the TE protein database [37]. We identified 691 Mb transposable elements (62.44% of the total sequence), including 580 Mb of LTR retrotransposons (52.43%) (Table 4).

**Table 4:** Overview of the annotation of the *P. tenuiflora* genome

		Total Length (bp)	% of Genome^a^	
Transposable	DNA	81,228,002	7.34	
Elements	LINE	33,892,567	3.06	
	SINE	154,638	0.01	
	LTR	580,518,664	52.43	
	Unknown	4,544,534	0.41	
	Total	691,362,441	62.44	
	Types/Copies	Total Length (bp)	% of Genome^a^	
Non-coding	miRNA (1376)	171,853	0.015522	
RNAs	tRNA (692)	52,086	0.004704	
	rRNA (68)	14,130	0.001276	
	snRNA (702)	83,103	0.007506	
Protein-coding	Predicted	Supported by	Supported by	Function
Genes		Transcriptome	Homologs	Assigned
	39,725	26,529	33,316	39,470 (99.4%)

^a^Assembled genome

### Annotation of protein-coding genes

A combined strategy (de novo-, homolog-, and RNA-seq-based predictions) was used to annotate protein-coding genes in the *P. tenuiflora* genome using the following software: Augustus (version 3.0.2) [40, 41], Genescan (version 1.0) [42], Geneid [43], GlimmerHMM (version 3.0.2) [44], and SNAP [45]. The homologous sequences of six species (*Zea mays*, *Sorghum bicolor*, *Brachypodium distachyon*, *Setaria italica*, *Arabidopsis thaliana*, and *Oryza sativa*) were aligned against the repeat-masked *P. tenuiflora* genome with TBLASTN (E-value ≤10–5) [46], and then Genewise software 2.2.0 was used to predict the gene models [47]. Two strategies were used to assemble the RNA-seq reads to the unique transcripts. First, we mapped the RNA-seq reads to the *P. tenuiflora* genome with Tophat 2.0.8 [48] and Cufflinks 2.1.1 software [49] (http://cufflinks.cbcb.umd.edu/). Afterward, we used Trinity [50] to assemble the RNA-seq reads, and then used PASA [51] (http://pasapipeline.github.io/) to improve the structure of the assembled genes. We generated non-redundant gene sets using EVidenceModeler (EVM) [52] via integrating gene prediction results of all methods. Finally, the predicted genes were filtered by three criteria: coding region length of ≤50 amino acids; FPKM < 5; and supported only by de novo strategy. Functions of the protein-coding genes were annotated by BLASTP program (best hit with E-value ≤1E-05) against three public protein databases: TrEMBL [53], Swiss-Prot, and NR. The protein domains were analyzed by InterProScan software (4.8) via searching against InterPro databases 29.0 [54], and the GO term information was collected from the InterPro annotation results [55]. Moreover, we also conducted KEGG annotation for all genes [56].

On the basis of *P. tenuiflora* genomic sequences, we predicted 39,725 protein-coding genes (Tables 5). Of the 39,725 predicted protein-coding genes, the protein sequences of 39,470 genes (99.4%) were similar to sequences of known proteins and could be annotated (Table 6). The average gene length was 2818.5 bp, and the average CDS length was 1082.0 bp. The average exon number per gene was 4.2, with an average exon length of 260.5 bp and average intron length of 550.8 bp (Table 5).

**Table 5:** General statistics for feature of predicted protein-coding genes of *P. tenuiflora* genome. Protein-coding genes were predicted through the annotation strategy of de novo prediction and evidence based on homology and transcriptome data. The gene model was integrated with EVM and corrected by PASA to obtain the final set of protein-coding genes

Gene set		Number	Average gene length (bp)	Average CDS length (bp)	Average exons per gene	Average exon length (bp)	Average intron length (bp)
De novo^*a*^	Augustus	59,267	1866.04	873.71	3.04	287.43	486.52
	GlimmerHMM	195,821	4538.43	540.7	2.16	250.76	3457.37
	SNAP	115,465	3464.46	615.94	2.8	220.02	1582.94
	Geneid	122,152	2958.40	684.27	3.08	222.01	1092.23
	Genscan	92,436	5507.46	609.14	2.96	205.68	2497.19
Homolog^b^	*Zea mays*	40,162	1988.08	978.69	3.32	294.72	434.93
	*Sorghum bicolor*	73,561	2000.94	1121.24	2.57	436.89	561.61
	*Brachypodium distachyon*	67,858	2097.32	1124.66	2.8	401.87	540.8
	*Setaria italica*	62,339	1568.92	826.04	2.68	308.16	442.05
	*Arabidopsis thaliana.*	43,096	1629.35	839.3	2.77	302.45	445.1
	*Oryza sativa*	76,835	1550.38	915.3	2.35	389.23	469.88
RNA-seq	Cufflinks^c^	62,560	5041.52	1845.64	5.54	333.32	704.38
	PASA	63,952	2292.77	934.6	3.9	239.86	468.9
EVM		66,649	2149.27	869.1	3.23	268.94	573.67
PASA-update		66,482	2122.71	871.22	3.22	270.77	564.36
Final set ^c^		39,725	2818.49	1081.99	4.15	260.54	550.76

^a^Statistics calculated from the gene set predicted from each method.

^b^Statistics calculated from the gene set predicted by homolog proteins from each species.

^c^Final results of *P. tenuiflora* genome

**Table 6:** Functional annotation of protein-coding genes against different databases. Gene functions were obtained from the best BLASTP hit

Database		Annotated Number	Annotated Percent (%)
NR		36,064	90.8
Swiss-Prot		25,684	64.7
KEGG		24,167	60.8
InterPro	^a^All	39,202	98.7
	Pfam	26,709	67.2
	GO	35,648	89.7
Total		39,470	99.4

^a^Combination of Pfam annotation and GO annotation

### Annotation of non-coding RNA

The tRNA genes were discovered with tRNAscan-SE software [57]. The rRNA, miRNA, and snRNA were predicted by INFERNAL software [58] against the Rfam database 9.1 [59]. We annotated non-coding RNA and identified 692 tRNAs, 68 rRNAs, 702 snRNAs, and 1376 microRNAs in the *P. tenuiflora* genome (Tables 4 and 7). The average lengths of microRNAs, tRNAs, rRNAs, and snRNAs were 124.89 bp, 75.27 bp, 207.79 bp, and 118.21 bp, respectively (Table 7). We deposited the genome sequence in the Genome Warehouse in National Genomics Data Center [60].

**Table 7:** Identification of non-coding RNAs of *P. tenuiflora* genome. The tRNAs were predicted by tRNAscan-SE software. The rRNA, miRNA and snRNA genes were extracted by INFERNAL software against the Rfam database

Type		Copy	Average length (bp)	Total length (bp)	% of genome
miRNA		1376	124.89	171,853	0.015522
tRNA		692	75.27	52,086	0.004704
rRNA	**rRNA**	68	207.79	14,130	0.001276
	18S	21	406.57	8538	0.000771
	28S	11	129.91	1429	0.000129
	5.8S	4	103.5	414	0.000037
	5S	32	117.16	3749	0.000339
snRNA	**snRNA**	702	118.21	83,103	0.007506
	CD-box	449	106.31	47,734	0.004311
	HACA-box	65	132.71	8626	0.000779
	splicing	188	141.41	26,585	0.002401

### Assessment of genome quality

We assessed genome quality using the following methods: Burrow-Wheeler Aligner (BWA), Core Eukaryotic Genes Mapping Approach (CEGMA), and Benchmarking Universal Single-Copy Orthologs (BUSCO). First, in order to assess the quality of genome assembly, we aligned the high-quality Illumina short reads to the assembly using BWA (http://bio-bwa.sourceforge.net, parameters ‘-o 1 -i 15’) [61]. According to BWA method, 87.41% of raw reads were mapped to the genome with 93.34% coverage (Table 8). Next, we used CEGMA and BUSCO to estimate completeness of the assembly. CEGMA is a set of conserved protein families for a wide range of eukaryotes, and is used to identify exon–intron structures of these conserved protein families in a new genomic sequence [62]. CEGMA analysis revealed 223 out of 248 ultraconserved eukaryotic genes (89.9%) in the *P. tenuiflora* genome indicating integrity for the core genes in the assembly (Table 9). Moreover, completeness of the assembly also was assessed using BUSCO [63] combined with TBLASTN [46], Augustus (version 3.0.2) [40, 41], and HMMER (version 3.1b2) [64]. The BUSCO analysis showed that our assemblies contained 86.8% complete and 1.7% fragmented embryophyta orthologs, suggesting that the assembly quality was high (Table 10).

**Table 8:** Genome coverage rate of raw data based on the BWA method. Mapping rate was generated by mapping raw reads to the *P. tenuiflora* genome to express the reliability of the genome coverage

		Percentage
Reads	Mapping rate (%)	87.41
Genome	Average sequencing depth	79.35
	Coverage (%)	93.34
	Coverage at least 4X (%)	90.11
	Coverage at least 10X (%)	86.97
	Coverage at least 20X (%)	82.46

**Table 9:** CEGMA analysis results of *P. tenuiflora* genome

Species	Complete		Complete + partial	
	Prots	% completeness	Prots	% completeness
*P. tenuiflora*	216	87.1	223	89.92

**Table 10:** BUSCO results of *P. tenuiflora* genome. C: Complete BUSCOs; S: Complete and single-copy BUSCOs; D: Complete and duplicated BUSCOs; F: Fragmented BUSCOs; M: Missing BUSCOs; n: Total BUSCO groups searched

Species	BUSCO notation assessment results
*P. tenuiflora*	C:86.8% [S:75.7%, D:11.1%], F:1.7%, M:11.5%, n:1440

### Utility and discussion

#### Description of database

The genome assembly of *P. tenuiflora* consisted of 14,036 contigs with a total size of 1.095 Gb. Finally, we assembled 2638 scaffolds with a total size of 1.107 Gb, contig N50 of 117 kb, and scaffold N50 of 950 kb. On the basis of *P. tenuiflora* genomic sequences, we predicted 39,725 protein-coding genes, and identified 692 tRNAs, 68 rRNAs, 702 snRNAs, 1376 microRNAs, and 691 Mb transposable elements. We assessed the quality and completeness of the assembled genome through BWA, CEGMA mapping, and BUSCO mapping (Tables 8, 9, 10). The results showed that our assembly had high quality. All raw data for genome assembly are deposited at NCBI. The genome sequence is deposited in the Genome Warehouse in National Genomics Data Center (https://bigd.big.ac.cn/gwh) (accession number GWHABHL00000000).

#### Significance of database

Halophytes belong to several families and are distributed among multiple clades; this broad distribution pattern suggests that the salinity tolerance mechanisms of halophytes have evolved numerous times or have multiple origins [2]. As a result, halophytes not only exhibit a wide range of salinity tolerance but have also evolved diverse molecular and physiological mechanisms for salinity tolerance [2]. This diversity complicates discovery of the salinity tolerance mechanisms of halophytes. To date, almost all known molecular mechanisms of salinity tolerance were characterized in glycophytes such as rice, wheat, and Arabidopsis [4–6]. Glycophytes only provide limited insights into mechanisms of salinity tolerance, and extreme halophytes may have enormous values for improving our understanding of salinity tolerance mechanisms. The genome sequence of extreme halophytes will unlock their molecular studies in salinity tolerance.

The Gramineae is an important plant group because it includes many important food crops, such as rice, wheat, maize, and barley. *P. tenuiflora*, an extreme Gramineae halophyte, is closely related to barley and wheat. Zhang et al. (2013) reported that *P. tenuiflora* can grow normally for 6 days under 900 mM NaCl and survive at pH 11 [23]. Wang et al. (2006) found that *P. tenuiflora* survived 670 mmol/L NaCl [13]. A growing number of molecular biology studies have focused on this species owing to its strong salinity tolerance and high genetic value for cereal improvement [16–28]. In the present study, we sequenced and assembled the *P. tenuiflora* genome (2n = 14, size 1.107 Gb). Our work may improve current understanding of salinity tolerance and provides genetic resources for cereal improvement.

## Data Availability

All raw data of genome sequencing are available at NCBI. Accession numbers for raw data of genome assembly are SRR7503009-SRR7503032, and SRP152905 and SRP239345 for transcriptional data. The genome sequence was deposited in the Genome Warehouse in National Genomics Data Center (https://bigd.big.ac.cn/gwh) [60], Beijing Institute of Genomics (BIG), Chinese Academy of Sciences, under accession number GWHABHL00000000 that is publicly accessible at https://bigd.big.ac.cn/search?dbId=gwh&q=GWHABHL00000000&page=1. Seeds of *P. tenuiflora* is available from the corresponsing author upon request.

## Abbreviations

BWA: Burrow-Wheeler aligner
CEGMA: Core eukaryotic genes mapping approach
BUSCO: Benchmarking universal single-copy orthologs
